# Disparity in clinical outcomes after cardiac surgery between private and public (NHS) payers in England

**DOI:** 10.1016/j.lanepe.2020.100003

**Published:** 2020-11-13

**Authors:** Umberto Benedetto, Arnaldo Dimagli, Ben Gibbison, Shubhra Sinha, Maria Pufulete, Daniel Fudulu, Lucia Cocomello, Alan J. Bryan, Sunil Ohri, Massimo Caputo, Graham Cooper, Tim Dong, Enoch Akowuah, Gianni D. Angelini

**Affiliations:** a Bristol Heart Institute, University of Bristol, Bristol Royal Infirmary, Bristol, UK; b Clinical Trials and Evaluation Unit, University of Bristol, Bristol, UK; c Wessex Cardiothoracic Centre, Southampton University Hospitals NHS Trust, Southampton, UK; d Department of Cardiothoracic Surgery, Northern General Hospital, Sheffield, UK; e James Cook Hospital, South Tees Hospitals NHS Foundation Trust, Middlesbrough, UK

## Abstract

**Background:**

There is little known about how payer status impacts clinical outcomes in a universal single-payer system such as the UK National Health Service (NHS). The aim of this study was to evaluate the relationship between payer status (private or public) and clinical outcomes following cardiac surgery from NHS providers in England.

**Methods:**

The National Adult Cardiac Surgery Audit (NACSA) registry was interrogated for patients who underwent adult cardiac surgery in England from 2009 to 2018. Information on socioeconomic status were provided by linkage with the Iteration of the English Indices of Deprivation (IoD). The primary outcome was in-hospital mortality. Secondary outcomes included incidence of in-hospital postoperative cerebrovascular accident (CVA), renal dialysis, sternal wound infection, and re-exploration. To assess whether payer status was an independent predictor of in-hospital mortality, binomial generalized linear mixed models (GLMM) were fitted along with 17 items forming the EuroSCORE and the IoD domains.

**Findings:**

The final sample consisted of 280,209 patients who underwent surgery in 31 NHS hospitals in England from 2009 to 2018. Of them, 5,967 (2.1%) and 274,242 (97.9%) were private and NHS payers respectively. Private payer status was associated with a lower risk of in-hospital mortality (OR 0.79; 95%CI 0.65 – 0.97;*P* = 0.026), CVA (OR 0.77; 95%CI 0.60 – 0.99; *P* = 0.039), need for re-exploration (OR 0.84; 95%CI 0.72 – 0.97; *P* = 0.017) and with non-significant lower risk of dialysis (OR 0.84; 95%CI 0.69 – 1.02; *P* = 0.074). Private payer status was found to be independently associated with lower risk of in-hospital mortality in the elective subgroup (OR 0.76; 95%CI 0.61 – 0.96; *P* = 0.020) but not in the non-elective subgroup (OR 1.01; 95%CI 0.64 – 1.58; *P* = 0.976).

**Interpretation:**

In conclusion, using a national database, we have found evidence of significant beneficial effect of payer status on hospital outcomes following cardiac surgery in favour of private payers regardless their socioeconomic factors.

**Research in context**

**Evidence before this study**

Universal health coverage represents the best way to guarantee health equity. However, inequalities within the health system still exist in those countries that have adopted a universal publicly financed health system. In particular, unequal distribution of social, environment and economic conditions has been recognized as sources of health inequalities. We searched PubMed using the following key words: “socio-economic inequality/status/disparity”, “primary payer status”, “health inequalities”, “cardiac surgery/heart surgery”. The literature search showed that research regarding the association between those factors and health inequalities in patients with cardiovascular disease in the UK remains scarce. Moreover, the effect of primary payer status on outcomes following cardiac surgery in the UK remains undefined.

**Added value of this study**

This analysis is based on a large data set comprising nearly a quarter of a million patients over a 10-year period and uses unbiased clinical outcomes (all-cause mortality) as primary outcomes and a standardized measure (the UK Index of Multiple Deprivation) to assess socioeconomic deprivation. The added value of this study is that in the UK, private payer status is associated with a lower risk of mortality and major complications in patients undergoing heart surgery with NHS providers. In particular, private payer status was associated with a risk reduction in mortality after controlling for case mix, variables related to the surgical procedures and neighbourhood socioeconomic status. The disadvantage for NHS payer status was more marked in elective patients, for those undergoing isolated CABG surgery and for those with most deprived neighbourhood socioeconomic status.

**Implications of all the available evidence**

The results of this study suggest that a complex interaction between socioeconomic and health system–related factors for patients undergoing cardiac surgery exists and further research is required to identify interventions to reduce health inequalities. Patients from the most deprived areas are more vulnerable but also more susceptible to the beneficial effect of preventive interventions which can ultimately reduce resource utilization. Finally**,** primary payer and socioeconomic status should be strongly considered during preoperative patient risk stratification in an effort to improve postoperative outcomes.

Alt-text: Unlabelled box

## 1 Introduction

Many healthcare systems distribute resources on the basis of equitable access to healthcare for people at the same risk [1]. However, health inequalities arising from the unequal distribution of social, environmental and economic conditions have been recognized in several clinical settings [2,3]. In particular, the influence of primary payer status (i.e. private or public) has become a central focus of modern healthcare system reforms and public scrutiny [2,3]. Acquired heart diseases remain the leading cause of death in Western countries [4]. Cardiac surgery constitutes the “gold standard” treatment in many cases and is the most frequently used and costly 'high-tech' procedures carried out. There is much interest as to whether primary payer and socioeconomic status affect clinical outcomes [5], [6], [7]. In the United States, where there is no universal healthcare program, people covered by government-assistance insurance programs (i.e., Medicaid and Medicare) have been shown to have worse clinical outcomes compared with privately insured patients. This occurs in multiple clinical settings, including cardiac surgery [2,[5], [6], [7]]. There is little known about how payer status impacts clinical outcomes in a universal single-payer system (also known as universal health coverage) such as the UK National Health Service (NHS) – financed entirely by the government through taxation. A small proportion of patients voluntarily subscribe to additional health insurance and have the option to access services privately provided by both public and private hospitals [8,9]. The outcomes of patients who pay for private healthcare within the NHS are of interest because they are treated by the same clinical teams as those who receive care funded by the government. Private payment allows the patients to access their surgery at a time of their choosing, with a surgeon of their choosing and access “cinderella” services such as single-rooms and enhanced menus. Therefore, understanding the outcomes of these patients could highlight complex socioeconomic and health-system related factors that might be targeted to improve clinical outcomes in patients undergoing cardiac surgery. The aim of this study was to evaluate the relationship between payer status (private or public) and clinical outcomes following cardiac surgery from NHS providers in the UK.

## 2 Methods

The study was approved by Health Research Authority (HRA) and Health and Care Research Wales (HCRW) (IRAS ID: 278,171).

### 2.1 Data and data management

The National Institute for Cardiovascular Outcomes Research (NICOR) National Adult Cardiac Surgery Audit (NACSA) registry was interrogated for patients who underwent adult cardiac surgery in England from 2009 to 2018. This registry prospectively collects demographic, as well as pre-, peri- and post-operative clinical information and mortality information for all major adult cardiac surgery procedures performed in England and its key function is benchmarking surgical practice. The risk model currently used to adjust for case-mix is the European System for Cardiac Operative Risk Evaluation (EuroSCORE) [10]. EuroSCORE is an operative mortality risk prediction model which takes into account 17 covariates encompassing patient-related, cardiac and operation-related characteristics.

Reproducible algorithms were applied to the database in order to clean the data [11]. There were 298,743 adult patients in the database reported as undergoing cardiac surgery in England from 2008 to 2019. Patients missing information on socioeconomic status (*n* = 12,947 patients) were excluded. We further excluded 5548 patients whose operation was carried out in exclusively private institutions (6 hospitals). Patients undergoing cardiac surgery in private hospitals in the England are highly selected and patients who present with complications postoperatively are commonly transferred to NHS hospitals, therefore making hospital outcomes difficult to interpret. Information on socioeconomic status were provided by linkage with the Iteration of the English Indices of Deprivation (IoD) [12]. Indices of Deprivation are the England Governments official measure of relative deprivation at a small local area level (Lower-layer Super Output Areas – equivalent to a neighbourhood) across England. The iteration is based on seven different domains of deprivation: Income Deprivation, Employment Deprivation, Education, Skills and Training Deprivation, Health Deprivation and Disability, Crime, Barriers to Housing and Services, Living Environment Deprivation. Combining information from the seven domains can be used to produces an overall relative measure of deprivation: The Index of Multiple Deprivation (IMD). The weight for each domain is derived from consideration of the academic literature on poverty and deprivation, as well as the levels of robustness of the indicators. The IoD ranks every neighbourhood in England from 1 (most deprived area) to 32,844 (least deprived area). Deciles are calculated by ranking the 32,844 neighbourhoods in England from most deprived to least deprived and dividing them into 10 equal groups.

### 2.2 Outcomes variables

The primary outcome was in-hospital mortality. Missing or conflicting data for in-hospital mortality were obtained via record linkage to the Office for National Statistics census database. Secondary outcomes included incidence of in-hospital postoperative cerebrovascular accident (CVA), renal dialysis, sternal wound infection, and re-exploration.

### 2.3 Statistical analysis

Categorical variables were summarized as counts and percentages. Continuous variables were summarized as median (interquartile range) or mean (SD). Baseline characteristics between the two groups were compared using standardized mean difference (SMD) and a value >0.2 was considered as indicative of meaningful imbalance. Standardization was based on the generalized linear mixed models (GLMM) analysis. To assess whether payer status (private or NHS) was an independent predictor of in-hospital mortality, binomial GLMMs were fitted. The 17 items forming the EuroSCORE and the seven IoD domains were included as fixed terms in the regression model. Outcomes were analysed separately using separate models. A clustering effect was anticipated for patients operated in the same hospital or by the same surgeon and therefore these two variables were included in the model as random intercepts. As surgical outcomes may have improved in the last decade, year of surgery was also included as random intercept. Random effects were reported as variance. One feature of the mixed effects model is that the variance of the random effect is directly interpretable. The marginal R-squared considers only the variance of the fixed effects, while the conditional R-squared takes both the fixed and random effects into account [13]. Three subgroup analyses were performed for the main outcome (mortality) to identify possible effect modifiers: (1) elective-vs non-elective (by excluding or limiting the analysis to patients with emergency surgery, unstable angina, acute endocarditis, critical preoperative state and ischaemic ventricular septal defect); (2) isolated coronary artery bypass grafting (CABG) procedures vs other than isolated CABG surgery; (3) least vs most deprived neighbourhood socioeconomic status (defined as IMD ≤5 vs IMD>5). We also tested the interaction between IMD decile and payer status in a fully mixed adjusted model checking for non-linearity by comparing a linear vs a natural spline term (*3 knots*). Effect estimates for fixed terms were reported as Odds ratio (OR) and relative 95% confidence. *P*-value <0.05 was considered significant in all the analysis. All analyses were performed in R version 4.0.0. lme4, lmerTest, sjPlot packages were used to fit and present GLMM results.

### 2.4 Role of funding source

The funder of the study had no role in study design, data collection, data analysis, data interpretation, or writing of the report. UB had full access to all the data in the study and UB, AD, BG, SS, MP, DF, LC, AB, SO, MC, GC, TD, EA, GDA had final responsibility for the decision to submit for publication.

## 3 Results

**Fig. 1:**
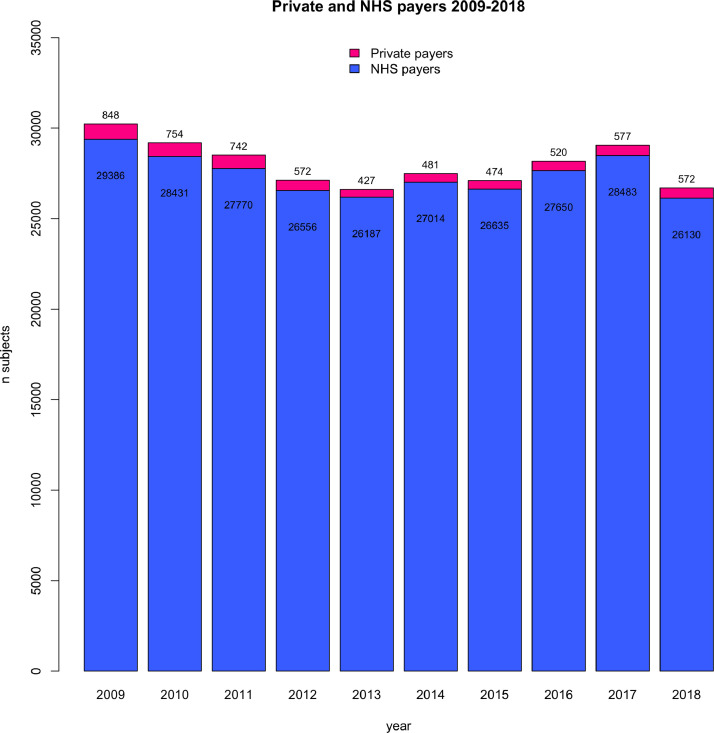
Bar plot with total number of NHS and private payers per year from 2009 to 2018.

**Fig. 2:**
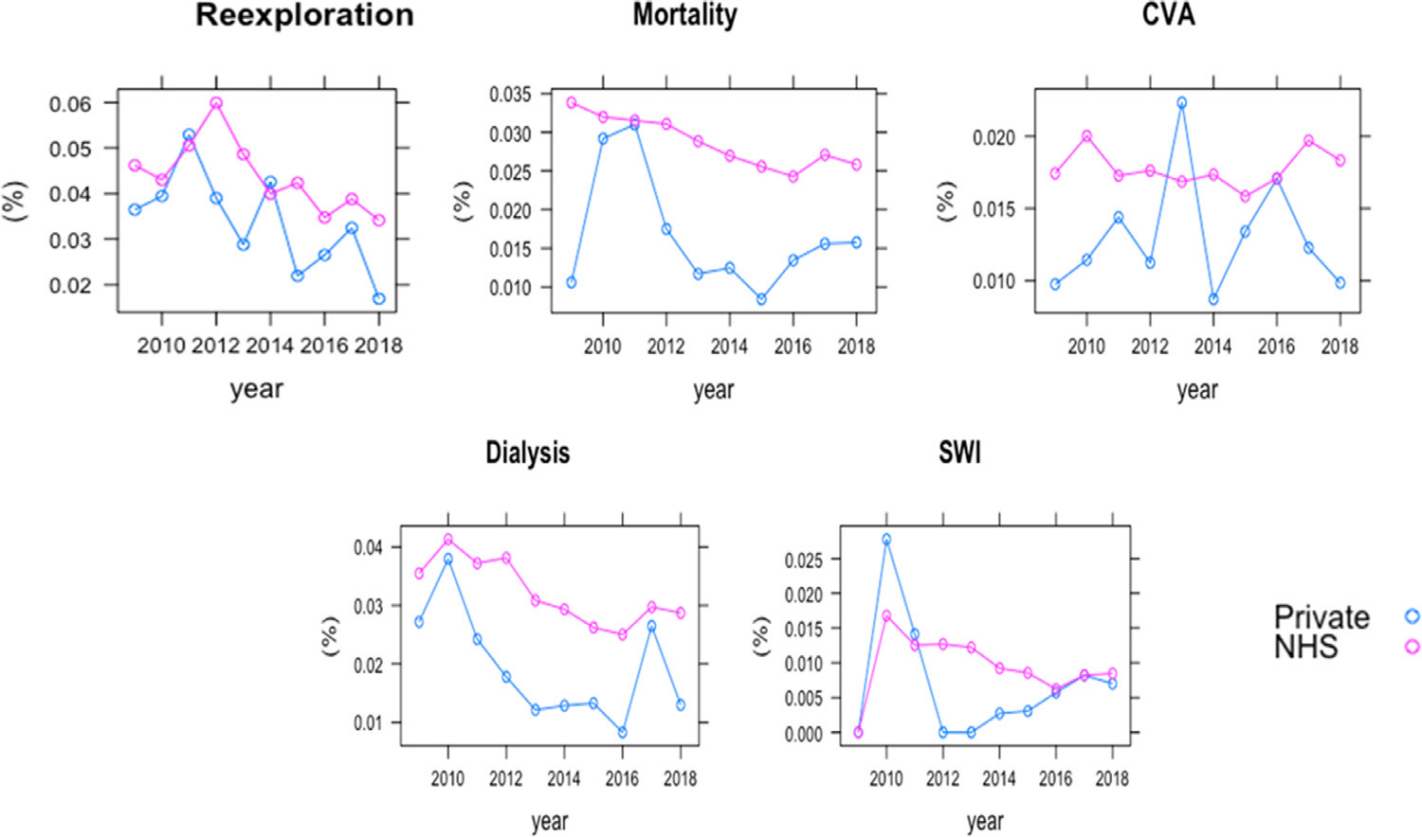
Dot plot with incidence of individual complications (re-exploration, mortality, cerebrovascular accidents [CVA], dialysis and sternal wound infection [SWI]) in NHS (red line) and private payers (blue line) per year. (For interpretation of the references to color in this figure legend, the reader is referred to the web version of this article.)

**Fig. 3:**
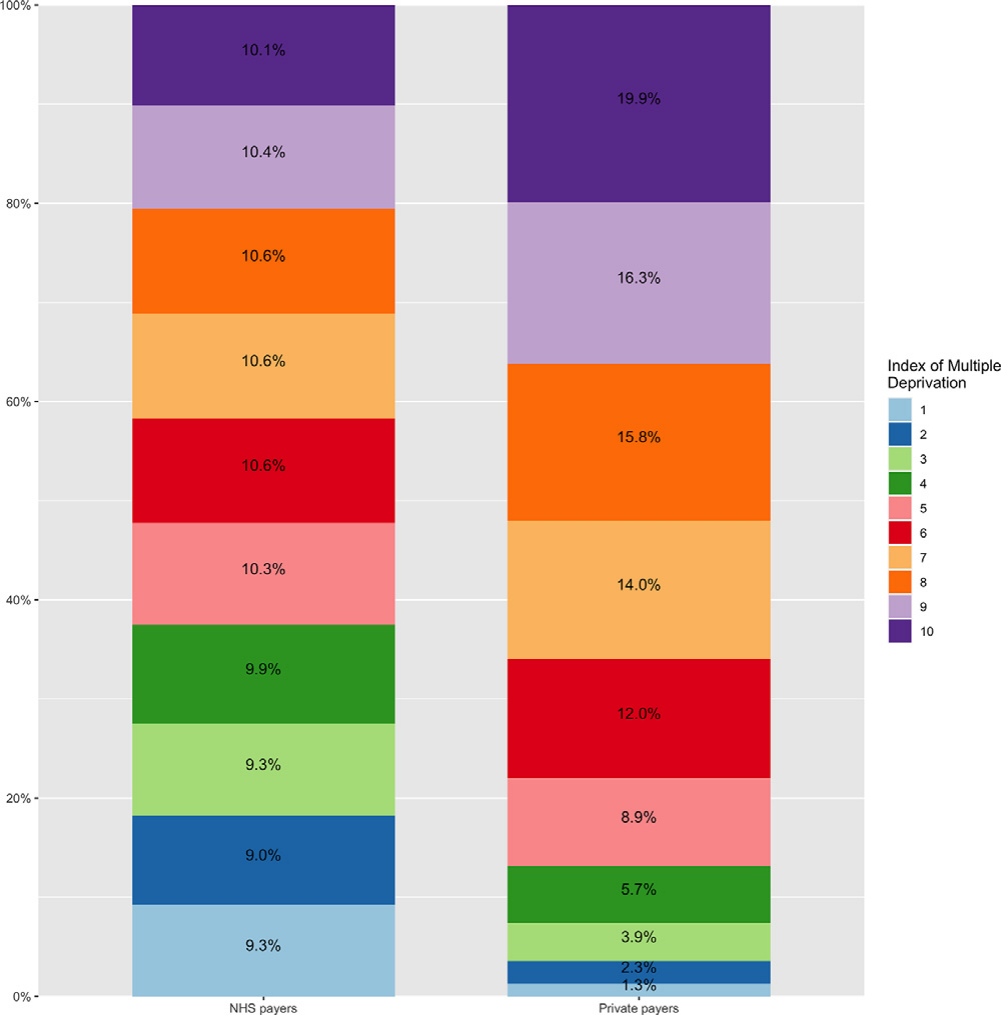
Distribution of Index of Multiple Deprivation (IMD) in NHS and private payers.

**Fig. 4:**
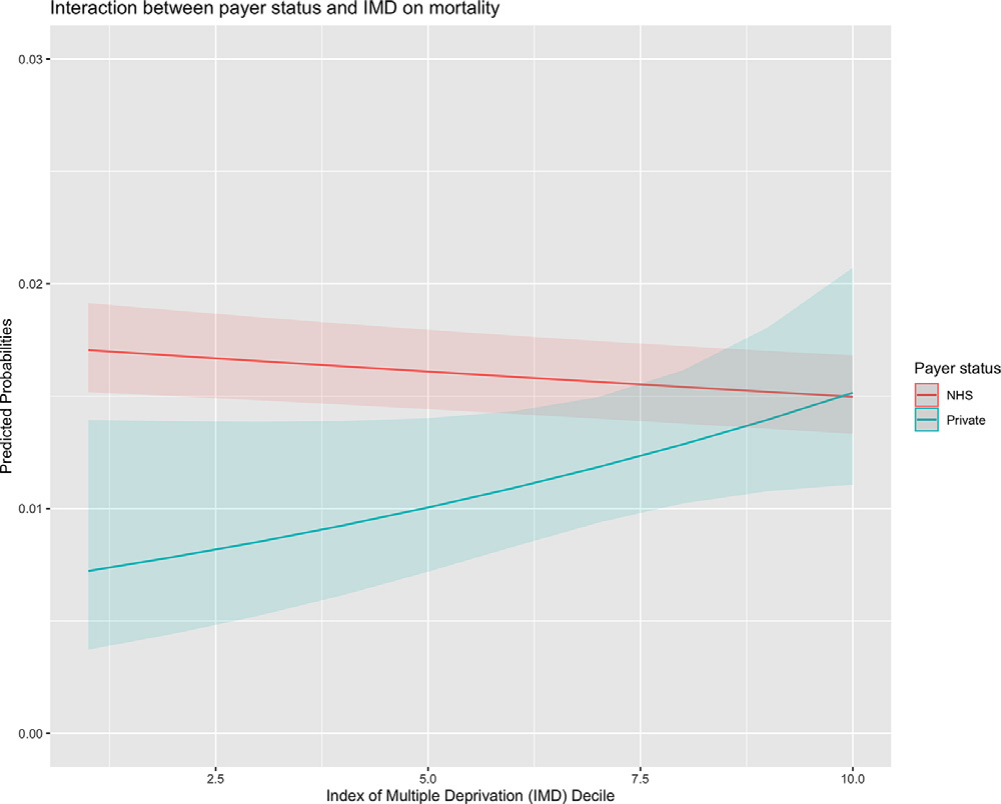
Interaction between payer status and Index of Multiple Deprivation (IMD).

The final sample consisted of 280,209 patients who underwent surgery in 31 NHS hospitals in England from 2009 to 2018. Of them, 5967 (2.1%) and 274,242 (97.9%) were private and NHS payers, respectively. The proportion of private payers ranged from 1.6% in 2013 to 2.8% in 2009 across years (Fig. 1) and from 0% to 7% across different hospitals (Supplementary Table 1). Patient characteristics and hospital outcomes are presented in Table 1. Private payers showed a tendency towards a lower risk profile including a lower incidence of recent myocardial infarction. When compared to NHS payers, private payers were associated with less deprived neighbourhood socioeconomic status (Fig. 2). When compared to NHS payers, private payers showed a lower incidence of in-hospital complications including mortality (1.7%. vs. 2.9%; *P*<0.001), cerebrovascular accident (1.3% vs. 1.8%, *P* = 0.007), surgical wound infection (0.5% vs 1.0%; *P* = 0.016), need for postoperative dialysis (2.0% vs 3.2%; *P*<0.001) and re-exploration (3.5% vs. 4.4%; *P*<0.001). This trend was consistent through the study period (Fig. 3). Table 2 summarizes multilevel, multivariable adjusted GLMM results. We then adjusted the effect of payer status for case-mix on outcomes related to the surgical procedures and neighbourhood socioeconomic status: private payer status was associated with a lower risk of in-hospital mortality (OR 0.79; 95%CI 0.65 – 0.97;*P* = 0.026), CVA (OR 0.77; 95%CI 0.60 – 0.99; *P* = 0.039), need for re-exploration (OR 0.84; 95%CI 0.72 – 0.97; *P* = 0.017) and with non-significant lower risk of dialysis (OR 0.84; 95%CI 0.69 – 1.02; *P* = 0.074). We found a more remarkable clustering effect related to individual surgeons (15% of surgeons associated with 45% relative risk increase of mortality) and individual hospitals (15% of hospital associated with 19% relative risk increase of mortality). When the analysis was stratified for elective vs non-elective subgroups, private payer status was found to be independently associated with lower risk of in-hospital mortality in the elective subgroup (OR 0.76; 95%CI 0.61 – 0.96; *P* = 0.020) but not in the non-elective subgroup (OR 1.01; 95%CI 0.64 – 1.58; *P* = 0.976) (Supplementary Table 2). When compared to NHS payer status, private payer status was independently associated with a lower risk of mortality in patients undergoing isolated CABG surgery OR (0.63; 95%CI 0.41 – 0.96; *P* = 0.033) while this trend was not significant for patients undergoing other than isolated CABG surgery (OR 0.86; 95%CI 0.68 – 1.08; *P* = 0.201) (Supplementary Table 3). Finally, the association between private payer status and lower risk of mortality was more marked in the least deprived patients (OR 0.42; 95%CI 0.24 – 0.74; *P* = 0.002) than in patients with most deprived neighbourhood socioeconomic status (OR 0.90; 95%CI 0.73 – 1.13; *P* = 0.370) (Supplementary Table 4). We found a significant association between payer status and IMD deciles (*P* = 0.04) with the largest difference in mortality between private and NHS payer at the lowest IMD deciles (Fig. 4).

**Table 1:** Patients characteristic and neighbourhood socioeconomic status in the National Adult Cardiac Surgery Audit registry in the 2009–2018 cohort (England).

	NHS payers	Private payers	SMD
**Number of patients**	274,242	5967	
***EuroSCORE risk factors***			
Age (mean (SD))	67.07 (11.77)	66.13 (11.30)	0.081
Female, n (%)	75,447 (27.5)	1286 (21.6)	0.139
Neurological dysfunction, n (%)	9986 (3.6)	126 (2.1)	0.092
Creatinine>200 mmol/l, n (%)	5728 (2.1)	44 (0.7)	0.115
Recent MI, n (%)	53,913 (19.7)	494 (8.3)	**0.333**
Critical preoperative state, n (%)	8691 (3.2)	71 (1.2)	0.136
Unstable angina, n (%)	12,458 (4.5)	111 (1.9)	0.153
Moderate LVEF, n (%)	60,461 (22.0)	1018 (17.1)	0.126
Poor LVEF, n (%)	13,732 (5.0)	209 (3.5)	0.075
Previous cardiac surgery, n (%)	13,553 (4.9)	361 (6.0)	0.049
Chronic pulmonary disease, n (%)	30,574 (11.1)	416 (7.0)	0.146
Extracardiac arteriopathy, n (%)	29,159 (10.6)	412 (6.9)	0.132
Pulmonary hypertension, n (%)	45,230 (16.5)	1055 (17.7)	0.032
Emergency, n (%)	10,648 (3.9)	88 (1.5)	0.150
Active endocarditis, n (%)	5448 (2.0)	66 (1.1)	0.071
Surgery on thoracic aorta, n (%)	17,408 (6.3)	403 (6.8)	0.016
Other than isolated CABG, n (%)	126,765 (46.2)	3139 (52.6)	0.128
Post infarct septal rupture, n (%)	599 (0.2)	2 (0.0)	0.052
***Indices of Deprivation (IoD)***			
Barriers to Housing and Services Decile (mean (SD))	5.63 (2.90)	4.97 (2.94)	**0.224**
Crime Decile (mean (SD))	5.77 (2.87)	7.05 (2.52)	**0.476**
Education and Skills Decile (mean (SD))	5.55 (2.82)	7.35 (2.37)	**0.687**
Employment Decile (mean (SD))	5.52 (2.82)	7.30 (2.35)	**0.686**
Health and Disability Decile (mean (SD))	5.56 (2.86)	7.39 (2.40)	**0.695**
Income Decile (mean (SD))	5.62 (2.82)	7.41 (2.28)	**0.698**
Living Environment Decile (mean (SD))	5.65 (2.86)	5.73 (2.86)	0.027
***In-hospital outcomes***			**P-value**
Mortality, n (%)	**7882 (2.9)**	**104 (1.7)**	**<0.001**
CVA, n (%)	**4326 (1.8)**	**68 (1.3)**	**0.007**
SWI, n (%)	**1473 (1.0)**	**17 (0.5)**	**0.016**
Dialysis, n (%)	**8033 (3.2)**	**111 (2.0)**	**<0.001**
*Re*-exploration, n (%)	**11,027 (4.4)**	**199 (3.5)**	**0.001**

CABG coronary artery bypass grafting; CVA cerebrovascular accidents; SWI sternal wound infection; LVEF left ventricular ejection fraction; MI myocardial infarction; NHS national health system; SMD standardized mean difference.

**Table 2:** Results of generalized linear mixed model (binomial) for outcomes of interest.

	Mortality			CVA			Dialysis			SWI			Re-exploration		
*Predictors*	*Odds Ratios*	*CI*	*p*	*Odds Ratios*	*CI*	*p*	*Odds Ratios*	*CI*	*p*	*Odds Ratios*	*CI*	*P*	*Odds Ratios*	*CI*	*p*
(Intercept)	0.00	0.00 – 0.00	**<0.001**	0.00	0.00 – 0.00	**<0.001**	0.00	0.00 – 0.00	**<0.001**	0.00	0.00 – 0.01	**<0.001**	0.01	0.01 – 0.01	**<0.001**
Age	1.04	1.04 – 1.05	**<0.001**	1.03	1.03 – 1.03	**<0.001**	1.03	1.03 – 1.04	**<0.001**	1.01	1.01 – 1.02	**<0.001**	1.02	1.01 – 1.02	**<0.001**
Female	1.44	1.36 – 1.51	**<0.001**	1.05	0.98 – 1.12	0.175	1.12	1.06 – 1.18	**<0.001**	1.06	0.94 – 1.19	0.344	0.78	0.74 – 0.81	**<0.001**
Neurological dysfunction	1.28	1.16 – 1.41	**<0.001**	1.75	1.56 – 1.97	**<0.001**	1.14	1.03 – 1.27	**0.011**	1.17	0.92 – 1.48	0.197	1.13	1.03 – 1.25	**0.008**
Creatinine>200 mmol/l	2.72	2.47 – 3.00	**<0.001**	1.33	1.13 – 1.57	**0.001**	7.26	6.69 – 7.87	**<0.001**	1.74	1.32 – 2.30	**<0.001**	1.74	1.57 – 1.92	**<0.001**
Recent MI	1.42	1.33 – 1.51	**<0.001**	1.06	0.96 – 1.17	0.222	1.42	1.33 – 1.52	**<0.001**	1.43	1.25 – 1.63	**<0.001**	1.51	1.43 – 1.59	**<0.001**
Critical preoperative state	2.11	1.94 – 2.31	**<0.001**	1.26	1.09 – 1.45	**0.002**	1.99	1.82 – 2.18	**<0.001**	1.07	0.83 – 1.38	0.593	1.34	1.22 – 1.48	**<0.001**
Unstable angina	1.20	1.10 – 1.32	**<0.001**	1.06	0.92 – 1.22	0.436	1.17	1.06 – 1.28	**0.001**	1.35	1.06 – 1.72	**0.014**	1.13	1.04 – 1.23	**0.006**
Moderate LVEF	1.55	1.47 – 1.64	**<0.001**	1.17	1.09 – 1.26	**<0.001**	1.48	1.40 – 1.56	**<0.001**	1.26	1.11 – 1.42	**<0.001**	1.08	1.03 – 1.14	**0.001**
Poor LVEF	2.99	2.77 – 3.23	**<0.001**	1.11	0.97 – 1.28	0.134	2.15	1.98 – 2.34	**<0.001**	1.38	1.12 – 1.71	**0.003**	1.20	1.10 – 1.30	**<0.001**
Previous cardiac surgery	3.09	2.88 – 3.32	**<0.001**	1.59	1.43 – 1.77	**<0.001**	2.02	1.86 – 2.18	**<0.001**	1.52	1.22 – 1.90	**<0.001**	1.20	1.11 – 1.30	**<0.001**
Chronic pulmonary disease	1.42	1.34 – 1.52	**<0.001**	1.13	1.03 – 1.24	**0.008**	1.37	1.28 – 1.46	**<0.001**	1.75	1.53 – 2.02	**<0.001**	0.95	0.89 – 1.01	0.083
Extracardiac arteriopathy	1.76	1.65 – 1.87	**<0.001**	1.66	1.53 – 1.80	**<0.001**	1.51	1.42 – 1.61	**<0.001**	1.28	1.10 – 1.49	**0.001**	1.04	0.98 – 1.10	0.224
Pulmonary hypertension	1.44	1.35 – 1.53	**<0.001**	1.05	0.96 – 1.15	0.284	1.47	1.38 – 1.57	**<0.001**	1.18	1.01 – 1.36	**0.032**	1.11	1.05 – 1.18	**<0.001**
Emergency	4.93	4.58 – 5.31	**<0.001**	2.99	2.69 – 3.33	**<0.001**	2.85	2.62 – 3.10	**<0.001**	1.17	0.90 – 1.52	0.248	1.57	1.44 – 1.71	**<0.001**
Active endocarditis	1.73	1.55 – 1.93	**<0.001**	1.22	1.04 – 1.43	**0.015**	1.69	1.51 – 1.89	**<0.001**	1.01	0.70 – 1.45	0.975	1.49	1.33 – 1.66	**<0.001**
Surgery on thoracic aorta	2.38	2.20 – 2.57	**<0.001**	2.70	2.46 – 2.97	**<0.001**	1.87	1.72 – 2.03	**<0.001**	1.09	0.86 – 1.38	0.475	1.58	1.47 – 1.69	**<0.001**
Other than isolated CABG	1.76	1.66 – 1.87	**<0.001**	1.83	1.70 – 1.98	**<0.001**	1.80	1.70 – 1.91	**<0.001**	0.95	0.84 – 1.08	0.478	2.00	1.91 – 2.10	**<0.001**
Post-infarct septal rupture	4.31	3.49 – 5.33	**<0.001**	1.08	0.65 – 1.81	0.767	3.01	2.36 – 3.84	**<0.001**	0.50	0.16 – 1.59	0.239	1.44	1.07 – 1.94	**0.017**
Private Vs NHS	0.79	0.65 – 0.97	**0.026**	0.77	0.60 – 0.99	**0.039**	0.84	0.69 – 1.02	0.074	0.73	0.46 – 1.18	0.203	0.84	0.72 – 0.97	**0.017**
Barriers to Housing and Services	1.00	0.99 – 1.01	0.369	0.99	0.98 – 1.00	0.128	0.99	0.98 – 1.00	0.176	1.00	0.98 – 1.02	0.983	1.00	0.99 – 1.00	0.338
Crime	0.98	0.97 – 1.00	**0.011**	1.00	0.98 – 1.01	0.783	1.00	0.99 – 1.01	0.660	0.99	0.97 – 1.02	0.670	1.00	0.99 – 1.01	0.825
Education and Skills	1.01	1.00 – 1.03	0.078	1.01	0.99 – 1.03	0.209	1.00	0.98 – 1.02	0.935	1.03	0.99 – 1.07	0.124	1.02	1.01 – 1.03	**0.005**
Employment	1.05	1.02 – 1.08	**0.002**	1.02	0.98 – 1.06	0.337	1.05	1.02 – 1.08	**0.001**	1.02	0.95 – 1.08	0.650	1.02	1.00 – 1.05	**0.045**
Health and Disability	0.98	0.96 – 1.00	**0.018**	1.00	0.97 – 1.02	0.770	0.98	0.96 – 1.00	**0.025**	0.99	0.94 – 1.03	0.505	0.99	0.97 – 1.00	0.141
Income	0.95	0.93 – 0.98	**0.001**	0.96	0.93 – 1.00	**0.043**	0.95	0.93 – 0.98	**0.001**	0.96	0.90 – 1.02	0.180	0.97	0.94 – 0.99	**0.004**
Living Environment	1.01	1.00 – 1.02	**0.012**	1.01	1.00 – 1.02	0.231	1.01	1.00 – 1.02	0.104	1.00	0.98 – 1.02	0.848	1.00	0.99 – 1.01	0.729
Random Effects															
σ^2^	3.29			3.29			3.29			3.29			3.29		
τ_00_	0.14 _Consultant_			0.12 _Consultant_			0.19 _Consultant_			0.49 _Consultant_			0.34 _Consultant_		
	0.03 _hospital_			0.88 _hospital_			0.64 _hospital_			4.11 _hospital_			0.15 _hospital_		
	0.01 _year_			0.00 _year_			0.03 _year_			0.05 _year_			0.03 _year_		
ICC	0.05			0.23			0.21			0.59			0.14		
N	649 _Consultant_			633 _Consultant_			628 _Consultant_			523 _Consultant_			636 _Consultant_		
	31 _hospital_			31 _hospital_			31 _hospital_			30 _hospital_			31 _hospital_		
	10 _year_			10 _year_			10 _year_			10 _year_			10 _year_		
Observations	280,117			248,741			254,846			152,748			257,041		
Marginal R^2^ / Conditional R^2^	0.228 / 0.270			0.100 / 0.309			0.147 / 0.322			0.020 / 0.594			0.058 / 0.188		

CABG coronary artery bypass grafting; CVA cerebrovascular accidents; ICC interclass correlation coefficient; LVEF left ventricular ejection fraction; MI myocardial infarction; NHS national health system; SMD standardized mean difference; SWI sternal wound infection.

## 4 Discussion

The main finding of the study was that in the UK, private payer status was associated with a lower risk of mortality and major complications in patients undergoing heart surgery with NHS providers. The primary analysis showed that private payer status was associated with 21% relative risk reduction in mortality after controlling for case mix, variables related to the surgical procedures and neighbourhood socioeconomic status. The disadvantage for NHS payer status was more marked in elective patients, for those undergoing isolated CABG surgery and for those with most deprived neighbourhood socioeconomic status.

Universal health coverage is a major health and political concern worldwide and has been described as the best way to achieve health equity. Recently, several countries have successfully switched to a publicly financed health system [4]. However, health inequalities persist even in countries with a publicly financed system, such as the UK. Wealth disparities and other social determinants are recognized causes of health inequalities, but health care-related factors may also have a role [8,9]. Overall, research regarding the association between health-care related factors and health inequalities in patients with cardiovascular disease in the UK remains scarce. Previous studies have suggested that an adverse association between increasing social deprivation and outcomes following cardiac surgery or heart transplant [[5], [6], [7],^14^,^15^]. However, these studies did not distinguish between effects due to payer status and effects due to socioeconomic deprivation. Because payer status and socioeconomic status tend to be highly correlated, this represents an important confounding factor.

Previously, the effect of payer status on outcomes following cardiac surgery in the UK remained undefined. We used a standardized measure of socioeconomic deprivation to adjust the effect of payer status but also a large number of other patient factors that cluster within socioeconomic status. We found evidence that private payer status was associated with increased survival and reduced incidence of complications. This association has a presumptive multi-factorial origin. An initial consideration is that NHS payers tended to present with a higher burden of comorbidities and more commonly underwent non-elective operations. For instance, NHS payers were more likely to undergo surgery in the context of a recent myocardial infarction. This might be related to the fact that private payers commonly access to the healthcare system at an earlier stage when compared to NHS payers. However, this observation also supports the hypothesis that private payers may be given priority on the waiting list over NHS payers. However, after adjusting for confounders including baseline clinical characteristics, payer status differences remained significant thus supporting a causal mechanism between health financing and clinical outcomes. Interestingly, the advantage from private payer status was significant amongst elective patients and those undergoing isolated CABG surgery. This observation could be explained by the fact that outcomes in high-risk setting are predominantly determined by the clinical presentations, while complex interaction between socioeconomic and health care-related factors may be relevant for patients at low risk. Second, the observed findings may be influenced by the bias amongst healthcare providers. For selected procedures, expert consultation at centers with higher operative volumes has been shown to improve postoperative outcomes. Private payers are likely referred to these high-volume centers while NHS payers may have little input in the selection of a surgeon. Moreover, primary care diagnostic patterns may differ as a function of payer status.  However, this analysis adjusted for the clustering effect related to individual surgeons and hospital and the association between payer status and outcomes remained significant. It should also be considered that private payers are operated on only by the responsible consultant surgeon and anaesthetist and they are not considered for training sessions. However, previous studies did not show any association between cases performed by trainees and increased risk of hospital mortality [16].

Remarkably, the advantage from private payer status was more relevant in patients with most deprived neighbourhood socioeconomic status after adjustment for case mix, procedure specific variables and clustering effect related to surgeons and hospitals, while this association was no longer significant in patients with least deprived neighbourhood socioeconomic status. We can speculate that patients with most deprived neighbourhood socioeconomic status are more likely to be exposed to communication failures and reduced levels of health literacy which may have an impact on their health outcomes. In this scenario, private payer status could counteract these barriers by promoting early access to healthcare system and by facilitating continuity of care which can ultimately result into improved clinical outcomes. Our results are supported by other reports based on outcomes from private healthcare systems, such as in the US. Recent efforts to examine the impact of primary payer and insurance status within surgical populations have focused on specific patient populations and surgical subspecialties and type of insurance was found to predict disease severity at the time of treatment [17,18].

### 4.1 Strength and limitations

The key strength of this study is that the analysis has been based on a large data set comprising nearly a quarter of a million patients over a 10-year period. It used unbiased clinical outcomes (all-cause mortality) as primary outcomes and a standardized measure (UK IMD) to assess socioeconomic deprivation. However, cause of death was not recorded in the NACSA database, meaning it is not possible to comment on mode of death and how this may have been influenced by payer status and socioeconomic deprivation. Notably, there was a large imbalance of group sizes and some hospitals had no private payers which may have affected estimates prediction.

We were also unable to distinguish between insured and self-funded private payers, although we believe that the last group should represent a very minority of patients. Also, it is possible that the ability to pay is simply a much more accurate indicator of personal socioeconomic status than area-based measures, and that therefore the effect captured in the analysis is still due to a more general effect of socioeconomic status. Regarding, the difference of the effect of payer status on elective vs non-elective patients, this could be due to the fact that surgeons could be more likely to perform elective high-risk surgery in private payers and therefore the magnitude of advantage from private payer status was actually larger than the one observed. Finally, the relative lack of confounding by the covariates does not truly establish a causal relationship between payer status and outcome.

## Conclusions

In conclusion, using a national database, we have found evidence of significant beneficial effect of payer status on hospital outcomes following cardiac surgery in favour of private payers regardless their socioeconomic factors. These results suggest that a complex interaction between socioeconomic and health system–related factors for patients undergoing cardiac surgery exists and further investigations are required to identify interventions which can reduce health inequalities. Patients from the most deprived areas are more vulnerable but also more susceptible to the beneficial effect of preventive interventions which can ultimately reduce resource utilization.

Although many of the strategies to tackle health inequalities lie outside the boundaries of healthcare services, healthcare organizations have significant opportunities to do far more to reduce inequalities in health. Strategies focusing on improving health equity may differ from those focusing only on the improvement of the average population health, as they are responsive only to the neediest ones and those with the highest risks of inequality. For instance, current benchmarking of cardiac units in UK could be stratified by socioeconomic status.

Finally, these results suggest, that primary payer and socioeconomic status should be strongly considered during preoperative patient risk stratification in an effort to improve postoperative outcomes.

## Declaration of Competing Interest

No conflicts of interest to disclose.

## Appendix Supplementary materials

Image, application 1
