# Supplementary material for: Disparity in clinical outcomes after cardiac surgery between private and public (NHS) payers in England
Source: Lancet Reg Health Eur. 2020 Nov 13;1:100003. doi: 10.1016/j.lanepe.2020.100003 (PMC8454835; doi:10.1016/j.lanepe.2020.100003)
Supplement: Supplementary file 1 [file mmc1.docx]

**Supplementary material**

**Disparity in clinical outcomes after cardiac surgery between private and public (NHS) payers in England.**

Umberto Benedetto et al.

**Content**

**Supplementary table 1.** Total number of cases and the number and proportion of private payers in each hospital.

**Supplementary Table 2.** Results of generalized linear mixed model (binomial) for the primary endpoint (mortality) among elective and non-elective patients.

**Supplementary Table 3.** Results of generalized linear mixed model (binomial) for the primary endpoint (mortality) among patients undergoing isolated CABG surgery or other than isolated CABG surgery.

**Supplementary Table 4.** Results of generalized linear mixed model (binomial) for the primary endpoint (mortality) among patients with most (IMD≤5) and least (IMD>5) deprived neighbourhood socioeconomic status.

**Supplementary table 1.** Total number of cases and the number and proportion of private payers in each hospital.

| Hospital | Total  Activity (n) | Private  Payers (n) | Private  payers (%) |
| --- | --- | --- | --- |
| #1 | 8802 | 72 | 0‧01 |
| #2 | 6924 | 454 | 0‧07 |
| #3 | 13814 | 58 | 0‧00 |
| #4 | 7029 | 202 | 0‧03 |
| #5 | 10537 | 163 | 0‧02 |
| #6 | 7362 | 78 | 0‧01 |
| #7 | 10785 | 95 | 0‧01 |
| #8 | 7057 | 132 | 0‧02 |
| #9 | 9063 | 231 | 0‧03 |
| #10 | 10466 | 0 | 0‧00 |
| #11 | 9586 | 360 | 0‧04 |
| #12 | 8779 | 354 | 0‧04 |
| #13 | 14198 | 475 | 0‧03 |
| #13 | 7309 | 5 | 0‧00 |
| #14 | 8730 | 154 | 0‧02 |
| #15 | 7857 | 47 | 0‧01 |
| #16 | 9501 | 6 | 0‧00 |
| #17 | 6803 | 30 | 0‧00 |
| #18 | 19468 | 1167 | 0‧06 |
| #19 | 6171 | 69 | 0‧01 |
| #20 | 7342 | 373 | 0‧05 |
| #21 | 6113 | 423 | 0‧07 |
| #22 | 12568 | 16 | 0‧00 |
| #23 | 9289 | 32 | 0‧00 |
| #24 | 339 | 18 | 0‧05 |
| #25 | 10720 | 55 | 0‧01 |
| #26 | 5623 | 54 | 0‧01 |
| #27 | 11184 | 231 | 0‧02 |
| #28 | 7432 | 164 | 0‧02 |
| #29 | 9735 | 0 | 0‧00 |
| #30 | 9623 | 449 | 0‧05 |

**Supplementary Table 2.** Results of generalized linear mixed model (binomial) for the primary endpoint (mortality) among elective and non-elective patients.

|  | **Death (Elective)** | | | **Death (Non-elective)** | | |
| --- | --- | --- | --- | --- | --- | --- |
| *Predictors* | *Odds Ratios* | *CI* | *p* | *Odds Ratios* | *CI* | *p* |
| (Intercept) | 0‧00 | 0‧00 – 0‧00 | **<0‧001** | 0‧00 | 0‧00 – 0‧00 | **<0‧001** |
| Age | 1‧05 | 1‧05 – 1‧06 | **<0‧001** | 1‧03 | 1‧03 – 1‧03 | **<0‧001** |
| Female | 1‧44 | 1‧36 – 1‧53 | **<0‧001** | 1‧39 | 1‧27 – 1‧51 | **<0‧001** |
| Neurological dysfunction | 1‧34 | 1‧18 – 1‧52 | **<0‧001** | 1‧17 | 1‧00 – 1‧35 | **0‧045** |
| Creatinine>200 mmol/l | 4‧02 | 3‧54 – 4‧56 | **<0‧001** | 1‧86 | 1‧62 – 2‧12 | **<0‧001** |
| Recent MI | 1‧49 | 1‧38 – 1‧62 | **<0‧001** | 1‧18 | 1‧05 – 1‧32 | **0‧004** |
| Moderate LVEF | 1‧59 | 1‧49 – 1‧70 | **<0‧001** | 1‧41 | 1‧27 – 1‧55 | **<0‧001** |
| Poor LVEF | 2‧93 | 2‧65 – 3‧23 | **<0‧001** | 2‧76 | 2‧44 – 3‧13 | **<0‧001** |
| Previous cardiac surgery | 3‧48 | 3‧19 – 3‧79 | **<0‧001** | 2‧48 | 2‧20 – 2‧79 | **<0‧001** |
| Chronic pulmonary disease | 1‧49 | 1‧38 – 1‧61 | **<0‧001** | 1‧28 | 1‧13 – 1‧44 | **<0‧001** |
| Extracardiac arteriopathy | 1‧83 | 1‧70 – 1‧96 | **<0‧001** | 1‧53 | 1‧37 – 1‧71 | **<0‧001** |
| Pulmonary hypertension | 1‧52 | 1‧41 – 1‧64 | **<0‧001** | 1‧22 | 1‧08 – 1‧37 | **0‧001** |
| Surgery on thoracic aorta | 2‧43 | 2‧21 – 2‧68 | **<0‧001** | 1‧98 | 1‧73 – 2‧26 | **<0‧001** |
| Other than isolated CABG | 1‧84 | 1‧71 – 1‧98 | **<0‧001** | 1‧49 | 1‧32 – 1‧68 | **<0‧001** |
| Private vs NHS | 0‧76 | 0‧61 – 0‧96 | **0‧020** | 1‧01 | 0‧64 – 1‧58 | 0‧976 |
| Barriers to Housing and Services Decile | 0‧99 | 0‧98 – 1‧00 | 0‧220 | 1‧00 | 0‧98 – 1‧02 | 0‧954 |
| Crime Decile | 0‧98 | 0‧97 – 1‧00 | **0‧028** | 0‧99 | 0‧97 – 1‧01 | 0‧153 |
| Education and Skills Decile | 1‧01 | 0‧99 – 1‧03 | 0‧221 | 1‧01 | 0‧99 – 1‧04 | 0‧282 |
| Employment Decile | 1‧04 | 1‧01 – 1‧08 | **0‧019** | 1‧05 | 1‧00 – 1‧11 | **0‧046** |
| Health and Disability Decile | 0‧98 | 0‧96 – 1‧00 | 0‧090 | 0‧97 | 0‧94 – 1‧01 | 0‧102 |
| Income Decile | 0‧96 | 0‧93 – 0‧99 | **0‧014** | 0‧95 | 0‧90 – 1‧00 | **0‧041** |
| Living Environment Decile | 1‧02 | 1‧01 – 1‧03 | **0‧004** | 1‧00 | 0‧99 – 1‧02 | 0‧614 |
| Critical preoperative state |  |  |  | 1‧87 | 1‧70 – 2‧06 | **<0‧001** |
| Unstable angina |  |  |  | 0‧99 | 0‧89 – 1‧10 | 0‧889 |
| Emergency |  |  |  | 3‧72 | 3‧39 – 4‧10 | **<0‧001** |
| Active endocarditis |  |  |  | 1‧43 | 1‧25 – 1‧64 | **<0‧001** |
| Post-infarct septal rupture |  |  |  | 3‧84 | 3‧12 – 4‧74 | **<0‧001** |
| **Random Effects** | | | | | | |
| σ^2^ | 3‧29 | | | 3‧29 | | |
| τ_00_ | 0‧16 _Consultant_ | | | 0‧13 _Consultant_ | | |
|  | 0‧03 _hospital_ | | | 0‧04 _hospital_ | | |
|  | 0‧02 _year_ | | | 0‧01 _year_ | | |
| ICC | 0‧06 | | | 0‧05 | | |
| N | 684 _Consultant_ | | | 628 _Consultant_ | | |
|  | 37 _hospital_ | | | 36 _hospital_ | | |
|  | 10 _year_ | | | 10 _year_ | | |
| Observations | 251563 | | | 28781 | | |
| Marginal R^2^ / Conditional R^2^ | 0‧214 / 0‧261 | | | 0‧264 / 0‧303 | | |

CABG coronary artery bypass grafting; CVA cerebrovascular accidents; ICC interclass correlation coefficient ;LVEF left ventricular ejection fraction; MI myocardial infarction; NHS national health system; SMD standardized mean difference; SWI sternal wound infection.

**Supplementary Table 3.** Results of generalized linear mixed model (binomial) for the primary endpoint (mortality) among patients undergoing isolated CABG surgery or other than isolated CABG surgery.

|  | **Death (isolated CABG)** | | | **Death (other than isolated CABG)** | | |
| --- | --- | --- | --- | --- | --- | --- |
| *Predictors* | *Odds Ratios* | *CI* | *p* | *Odds Ratios* | *CI* | *p* |
| (Intercept) | 0‧00 | 0‧00 – 0‧00 | **<0‧001** | 0‧00 | 0‧00 – 0‧00 | **<0‧001** |
| Age | 1‧05 | 1‧05 – 1‧06 | **<0‧001** | 1‧04 | 1‧04 – 1‧04 | **<0‧001** |
| Female | 1‧66 | 1‧52 – 1‧82 | **<0‧001** | 1‧35 | 1‧27 – 1‧43 | **<0‧001** |
| Neurological dysfunction | 1‧48 | 1‧23 – 1‧76 | **<0‧001** | 1‧20 | 1‧07 – 1‧35 | **0‧003** |
| Creatinine >200 mmol/l | 3‧47 | 2‧95 – 4‧08 | **<0‧001** | 2‧49 | 2‧21 – 2‧81 | **<0‧001** |
| Recent MI | 1‧19 | 1‧09 – 1‧30 | **<0‧001** | 1‧81 | 1‧64 – 2‧00 | **<0‧001** |
| Critical preoperative state | 2‧21 | 1‧94 – 2‧53 | **<0‧001** | 1‧92 | 1‧70 – 2‧16 | **<0‧001** |
| Unstable angina | 1‧14 | 1‧00 – 1‧29 | 0‧054 | 1‧26 | 1‧10 – 1‧43 | **0‧001** |
| Moderate LVEF | 1‧67 | 1‧52 – 1‧83 | **<0‧001** | 1‧48 | 1‧38 – 1‧59 | **<0‧001** |
| Poor LVEF | 3‧73 | 3‧31 – 4‧21 | **<0‧001** | 2‧51 | 2‧27 – 2‧78 | **<0‧001** |
| Previous cardiac surgery | 3‧79 | 3‧20 – 4‧49 | **<0‧001** | 2‧96 | 2‧73 – 3‧20 | **<0‧001** |
| Chronic pulmonary disease | 1‧37 | 1‧22 – 1‧53 | **<0‧001** | 1‧46 | 1‧35 – 1‧58 | **<0‧001** |
| Extracardiac arteriopathy | 1‧73 | 1‧57 – 1‧91 | **<0‧001** | 1‧76 | 1‧63 – 1‧91 | **<0‧001** |
| Pulmonary hypertension | 1‧35 | 1‧18 – 1‧54 | **<0‧001** | 1‧47 | 1‧37 – 1‧59 | **<0‧001** |
| Emergency | 5‧88 | 5‧18 – 6‧68 | **<0‧001** | 4‧32 | 3‧94 – 4‧75 | **<0‧001** |
| Private vs NHS | 0‧63 | 0‧41 – 0‧96 | **0‧033** | 0‧86 | 0‧68 – 1‧08 | 0‧201 |
| Barriers to Housing and Services Decile | 1‧00 | 0‧98 – 1‧02 | 0‧941 | 0‧99 | 0‧98 – 1‧01 | 0‧278 |
| Crime Decile | 0‧99 | 0‧97 – 1‧01 | 0‧420 | 0‧98 | 0‧97 – 1‧00 | **0‧010** |
| Education and Skills Decile | 1‧05 | 1‧02 – 1‧08 | **<0‧001** | 1‧00 | 0‧98 – 1‧02 | 0‧908 |
| Employment Decile | 1‧05 | 1‧00 – 1‧10 | 0‧072 | 1‧05 | 1‧01 – 1‧08 | **0‧014** |
| Health and Disability Decile | 0‧98 | 0‧95 – 1‧01 | 0‧223 | 0‧98 | 0‧95 – 1‧00 | **0‧037** |
| Income Decile | 0‧91 | 0‧86 – 0‧95 | **<0‧001** | 0‧98 | 0‧94 – 1‧01 | 0‧182 |
| Living Environment Decile | 1‧02 | 1‧01 – 1‧04 | **0‧010** | 1‧01 | 1‧00 – 1‧02 | 0‧175 |
| Active endocarditis |  |  |  | 1‧85 | 1‧66 – 2‧07 | **<0‧001** |
| Surgery on thoracic aorta |  |  |  | 2‧50 | 2‧30 – 2‧70 | **<0‧001** |
| Post-infarct septal defect |  |  |  | 2‧18 | 1‧31 – 3‧63 | **0‧003** |
| **Random Effects** | | | | | | |
| σ^2^ | 3‧29 | | | 3‧29 | | |
| τ_00_ | 0‧14 _Consultant_ | | | 0‧17 _Consultant_ | | |
|  | 0‧03 _hospital_ | | | 0‧03 _hospital_ | | |
|  | 0‧02 _year_ | | | 0‧01 _year_ | | |
| ICC | 0‧06 | | | 0‧06 | | |
| N | 629 _Consultant_ | | | 624 _Consultant_ | | |
|  | 31 _hospital_ | | | 31 _hospital_ | | |
|  | 10 _year_ | | | 10 _year_ | | |
| Observations | 149824 | | | 129849 | | |
| Marginal R^2^ / Conditional R^2^ | 0‧201 / 0‧246 | | | 0‧197 / 0‧246 | | |

CABG coronary artery bypass grafting; CVA cerebrovascular accidents; ICC interclass correlation coefficient ;LVEF left ventricular ejection fraction; MI myocardial infarction; NHS national health system; SMD standardized mean difference; SWI sternal wound infection.

**Supplementary Table 4.** Results of generalized linear mixed model (binomial) for the primary endpoint (mortality) among patients with most (IMD≤5) and least (IMD>5) deprived neighbourhood socioeconomic status.

|  | **Death (IMD**5) | | | **Death (IMD**$>$5) | | |
| --- | --- | --- | --- | --- | --- | --- |
| *Predictors* | *Odds Ratios* | *CI* | *p* | *Odds Ratios* | *CI* | *p* |
| (Intercept) | 0‧00 | 0‧00 – 0‧00 | **<0‧001** | 0‧00 | 0‧00 – 0‧00 | **<0‧001** |
| Age | 1‧04 | 1‧04 – 1‧04 | **<0‧001** | 1‧05 | 1‧04 – 1‧05 | **<0‧001** |
| Female | 1‧43 | 1‧33 – 1‧54 | **<0‧001** | 1‧46 | 1‧36 – 1‧56 | **<0‧001** |
| Neurological dysfunction | 1‧28 | 1‧11 – 1‧46 | **<0‧001** | 1‧29 | 1‧12 – 1‧49 | **0‧001** |
| Creatinine>200 mmol/l | 2‧37 | 2‧07 – 2‧72 | **<0‧001** | 3‧17 | 2‧77 – 3‧63 | **<0‧001** |
| Recent MI | 1‧42 | 1‧29 – 1‧56 | **<0‧001** | 1‧43 | 1‧30 – 1‧57 | **<0‧001** |
| Critical preoperative state | 2‧02 | 1‧79 – 2‧29 | **<0‧001** | 2‧18 | 1‧93 – 2‧46 | **<0‧001** |
| Unstable angina | 1‧28 | 1‧13 – 1‧44 | **<0‧001** | 1‧14 | 1‧01 – 1‧30 | **0‧039** |
| Moderate LVEF | 1‧52 | 1‧40 – 1‧64 | **<0‧001** | 1‧59 | 1‧47 – 1‧71 | **<0‧001** |
| PoorLVEF | 2‧94 | 2‧64 – 3‧27 | **<0‧001** | 3‧05 | 2‧73 – 3‧40 | **<0‧001** |
| Previous cardiac surgery | 3‧00 | 2‧70 – 3‧33 | **<0‧001** | 3‧24 | 2‧94 – 3‧57 | **<0‧001** |
| Chronic pulmonary disease | 1‧39 | 1‧27 – 1‧52 | **<0‧001** | 1‧49 | 1‧36 – 1‧63 | **<0‧001** |
| Extracardiac arteriopathy | 1‧76 | 1‧61 – 1‧92 | **<0‧001** | 1‧76 | 1‧61 – 1‧92 | **<0‧001** |
| Pulmonary hypertension | 1‧39 | 1‧27 – 1‧53 | **<0‧001** | 1‧43 | 1‧32 – 1‧57 | **<0‧001** |
| Emergency | 5‧14 | 4‧62 – 5‧72 | **<0‧001** | 4‧75 | 4‧28 – 5‧27 | **<0‧001** |
| Active endocarditis | 1‧89 | 1‧61 – 2‧21 | **<0‧001** | 1‧58 | 1‧35 – 1‧85 | **<0‧001** |
| Surgery on thoracic aorta | 2‧31 | 2‧06 – 2‧58 | **<0‧001** | 2‧41 | 2‧18 – 2‧68 | **<0‧001** |
| Other than isolated CABG | 1‧73 | 1‧59 – 1‧89 | **<0‧001** | 1‧78 | 1‧63 – 1‧94 | **<0‧001** |
| Post-infarct septal defect | 3‧88 | 2‧88 – 5‧22 | **<0‧001** | 4‧82 | 3‧56 – 6‧53 | **<0‧001** |
| Private vs NHS | 0‧42 | 0‧24 – 0‧74 | **0‧002** | 0‧90 | 0‧73 – 1‧13 | 0‧370 |
| **Random Effects** | | | | | | |
| σ^2^ | 3‧29 | | | 3‧29 | | |
| τ_00_ | 0‧12 _Consultant_ | | | 0‧16 _Consultant_ | | |
|  | 0‧03 _hospital_ | | | 0‧04 _hospital_ | | |
|  | 0‧01 _year_ | | | 0‧02 _year_ | | |
| ICC | 0‧05 | | | 0‧06 | | |
| N | 632 _Consultant_ | | | 631 _Consultant_ | | |
|  | 31 _hospital_ | | | 31 _hospital_ | | |
|  | 10 _year_ | | | 10 _year_ | | |
| Observations | 132257 | | | 147860 | | |
| Marginal R^2^ / Conditional R^2^ | 0‧225 / 0‧260 | | | 0‧231 / 0‧280 | | |

CABG coronary artery bypass grafting; CVA cerebrovascular accidents; ICC interclass correlation coefficient ;LVEF left ventricular ejection fraction; MI myocardial infarction; NHS national health system; SMD standardized mean difference; SWI sternal wound infection.
